# Primary Aldosteronism More Prevalent in Patients With Cardioembolic Stroke and Atrial Fibrillation

**DOI:** 10.3389/fendo.2022.869980

**Published:** 2022-04-19

**Authors:** Van Nguyen, Tian Ming Tu, Marlie Jane B. Mamauag, Jovan Lai, Seyed Ehsan Saffari, Tar Choon Aw, Lizhen Ong, Roger S. Y. Foo, Siang Chew Chai, Shaun Fones, Meifen Zhang, Troy H. Puar

**Affiliations:** ^1^ Doctor of Medicine Programme, Duke National University of Singapore (NUS) Medical School, Singapore, Singapore; ^2^ Department of Neurology, National Neuroscience Institute, Singapore, Singapore; ^3^ Department of Medicine, Neurology Division, Changi General Hospital (CGH), Singapore, Singapore; ^4^ Bachelor of Medicine, Bachelor of Surgery Programme, Yong Loo Lin School of Medicine, Singapore, Singapore; ^5^ Health Services and Systems Research, Duke–National University of Singapore (NUS) Medical School, Singapore, Singapore; ^6^ Department of Laboratory Medicine, CGH, Singapore, Singapore; ^7^ Department of Laboratory Medicine, National University Health System (NUHS), Singapore, Singapore; ^8^ Genome Institute of Singapore, Singapore, Singapore; ^9^ Cardiovascular Research Institute, NUHS, Singapore, Singapore; ^10^ Department of Cardiology, CGH, Singapore, Singapore; ^11^ Department of Endocrinology, CGH, Singapore, Singapore

**Keywords:** hyperaldosteronism, cerebrovascular accident, transient ischaemic attack, atrial fibrillation, secondary hypertension, endocrine hypertension

## Abstract

**Background:**

Primary aldosteronism (PA) is the most common cause of secondary hypertension, and patients are at an increased risk of atrial fibrillation (AF) and stroke. We assessed the prevalence of PA in patients with recent stroke.

**Methods:**

We recruited 300 patients admitted to an acute stroke unit with diagnosis of cerebrovascular accident (haemorrhagic/ischaemic) or transient ischaemic attack. Three months post-stroke, plasma renin and aldosterone were measured. Patients with an elevated aldosterone–renin ratio proceeded to the confirmatory saline loading test.

**Results:**

Twenty-six of 192 (14%) patients had an elevated aldosterone–renin ratio. Three of 14 patients who proceeded to saline loading were confirmed with PA (post-saline aldosterone >138 pmol/l). Another three patients were classified as confirmed/likely PA based on the markedly elevated aldosterone–renin ratio and clinical characteristics. The overall prevalence of PA amongst stroke patients with hypertension was 4.0% (95% confidence interval (CI): 0.9%–7.1%). Prevalence of PA was higher amongst patients with cardioembolic stroke, 11% (95% CI: 1.3%–33%), resistant hypertension, 11% (95% CI: 0.3%–48%), and hypertension and AF, 30% (95%CI: 6.7%–65%). If only young patients or those with hypokalaemia were screened for PA, half of our patients with PA would not have been diagnosed. Our decision tree identified that stroke patients with AF and diastolic blood pressure ≥83mmHg were most likely to have PA.

**Conclusion:**

We found that amongst hypertensive patients with stroke, PA was more prevalent in those with AF, or cardioembolic stroke. Screening for PA should be considered for all patients with stroke.

**Graphical Abstract:**
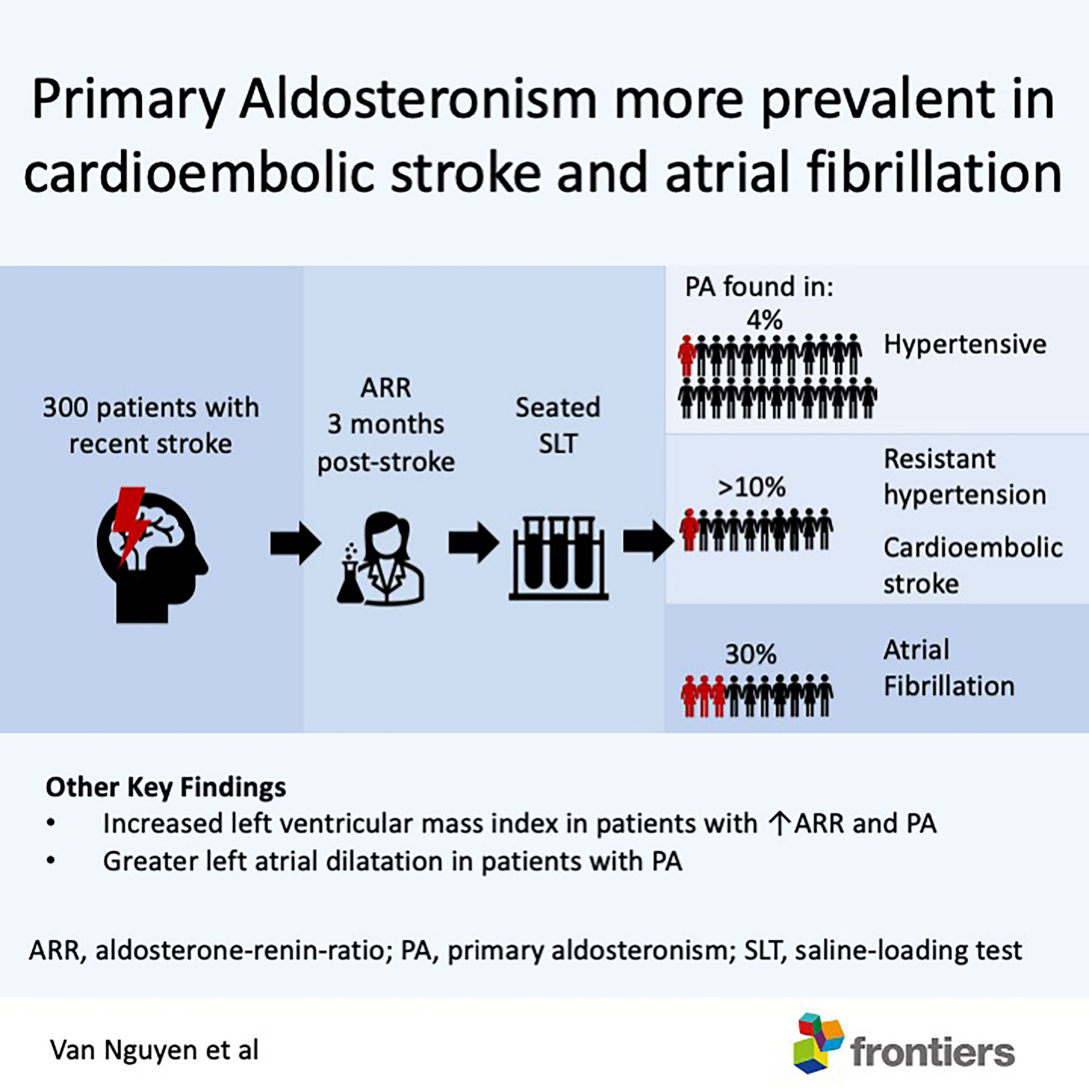
Amongst patients with recent stroke, primary aldosteronism was more prevalent in those with atrial fibrillation, cardio-embolic stroke and resistant hypertension. Patients with primary aldosteronism had greater left ventricular mass and larger left atrial volume. Patients with good functional recovery after a stroke should be screened for primary aldosteronism.

## Introduction

Primary aldosteronism (PA) is the most common treatable cause of hypertension, affecting 5% of all patients with hypertension (1), and up to 25% amongst those with severe or resistant hypertension (2). Compared to patients with essential hypertension of similar blood pressure (BP), patients with PA are at a greater risk of renal and cardiovascular events (3, 4), due to the direct deleterious effects of aldosterone. Since hypertension is the most important risk factor for stroke (5), PA is likely as prevalent amongst patients with stroke.

Importantly, PA has been associated with increased risk of atrial fibrillation (AF), due to the presence of mineralocorticoid receptors on the myocardium (6, 7). A recent study by Seccia and colleagues found a prevalence of PA in 42% of hypertensive patients with unexplained AF, which may further compound the risk of stroke in PA (8). While hypertension is the main risk factor for both haemorrhagic stroke, and ischaemic strokes from large or small vessel disease (9), AF is the major risk factor for cardioembolic strokes (10).

Treatment of PA can ameliorate the risk of cardiovascular events, renal disease, and even improve quality of life, which underlies the importance of diagnosing and treating PA (11). Furthermore, patients with unilateral PA may be cured with surgery, leading to better outcomes compared with medical therapy (12, 13). There is currently no neurology consensus on which patients with stroke should be screened for secondary causes of hypertension. While some guidelines recommend screening young patients with stroke, the definition of a young age differs between guidelines (14). Current Endocrine Society guidelines recommend screening for PA in patients with severe or resistant hypertension, and those with family history of stroke below 40, but do not have a recommendation for patients with stroke (15). Hence, we hypothesized that PA is prevalent amongst patients with stroke and conducted a prospective trial in patients admitted with an acute stroke to assess the prevalence of PA.

## Methods

We prospectively recruited 300 patients admitted to the acute stroke unit of Changi General Hospital, Singapore, between August 2018 and October 2020. Inclusion criteria were age 21 to 80 years, diagnosis of transient ischaemic attack (TIA), or ischaemic/haemorrhagic acute cerebrovascular accident (CVA). Exclusion criteria were limited life expectancy (e.g. terminal illness), pregnancy, impaired renal function (estimated glomerular filtration rate (eGFR) <45ml/min), and impaired cardiac function (ejection fraction 45% or lower). Chronic kidney disease was defined as eGFR <60 ml/min. Dyslipidaemia was defined as LDL ≥1.8 mmol/l, or use of lipid-lowering medications. The study was approved by the local ethics committee, registered with Clinicaltrials.gov (NCT03789357), and informed consent was obtained in all patients. Baseline characteristics such as demographics, medical history, drug information, and comorbidities were collected.

### Stroke Diagnosis and Management

All patients were managed for stroke in accordance with best clinical practice (16). Patients underwent further tests to determine the aetiology of stroke, which included computed tomography or magnetic resonance imaging of the brain, ultrasound of carotid arteries, 2-dimensional transthoracic echocardiogram (2DE), and 24-h Holter monitoring as indicated. All of the above test results were reviewed independently by two investigators (VN, TT), and subtypes of stroke were classified based on the Trial of Org 10172 in Acute Stroke Treatment (TOAST) Classification (17). Functional recovery status at 3 months or more were determined using the Modified Rankin Scale (MRS) (18).

### BP Medications and Tests for PA

In the acute and post-acute management, choice of antihypertensive medications and BP targets were left to the managing neurologist, according to current treatment guidelines. Patients with newly diagnosed hypertension were started on first-line antihypertensive medications, usually angiotensin-converting enzyme (ACE) inhibitors or calcium-channel blockers. Two to four months post-stroke, all patients had a morning seated blood test for plasma aldosterone concentration (PAC), plasma renin activity (PRA), and renal function. All antihypertensive medications were continued prior to the screening test, and fifteen patients with hypokalaemia (serum potassium <3.5 mmol/l) prior to the screening test were prescribed potassium supplementation. Clinic BP was taken in the seated position after 5 min of rest. Resistant hypertension was defined as clinic systolic BP ≥140 mmHg, or diastolic BP ≥90 mmHg, while on three antihypertensive medications. The screening test was positive if the aldosterone–renin ratio (ARR) was greater than 277 (pmol/l per ng/ml/h). Patients with a positive screening test underwent a confirmatory seated saline-loading test (SLT). Patients were confirmed with PA if post-saline PAC was >138 pmol/l or had spontaneous hypokalaemia with undetectable plasma renin activity (PRA) and PAC was >277 pmol/l (15). PAC and PRA were analysed by Mayo Clinic Laboratories, Rochester, MN, USA, for determination using LC–MS/MS, and the reference ranges were 0.6–3.0 ng/ml/h and 581 pmol/l or less, respectively, with the lowest level of detection 0.6 ng/ml/h and 110 pmol/l, respectively. For calculation of ARR, PRA was taken to be 0.6 ng/ml/h in those with undetectable PRA. Patients with PA were managed by an endocrinologist in accordance with the Endocrine Society guidelines.

### Statistical Analysis

Statistical analysis was conducted using R (A language and environment for statistical computing. R Foundation for Statistical Computing, Vienna, Austria. URL: https://www.R–project.org/). Continuous variables were expressed as a mean and standard deviation (SD) or median, minimum, and maximum or first and third quartile as appropriate and analysed using the independent-sample *t*-test or Mann–Whitney U test, depending on whether normality assumption was tenable. Categorical variables were presented as number (percent) and compared using the chi-squared test or Fisher exact test (where appropriate). The primary outcome was to determine the prevalence of PA in our cohort of patients with stroke. Secondary outcomes included the prevalence of PA in certain subgroups, and differences in characteristics of patients: those with PA versus those without PA, those with positive ARR versus negative ARR. A decision tree analysis *via* recursive partitioning for the classification method was performed to investigate how the baseline characteristics can help in discriminating patients with and without PA. Significance level was set at *p* value < 0.05.

## Results

**Figure 1:**
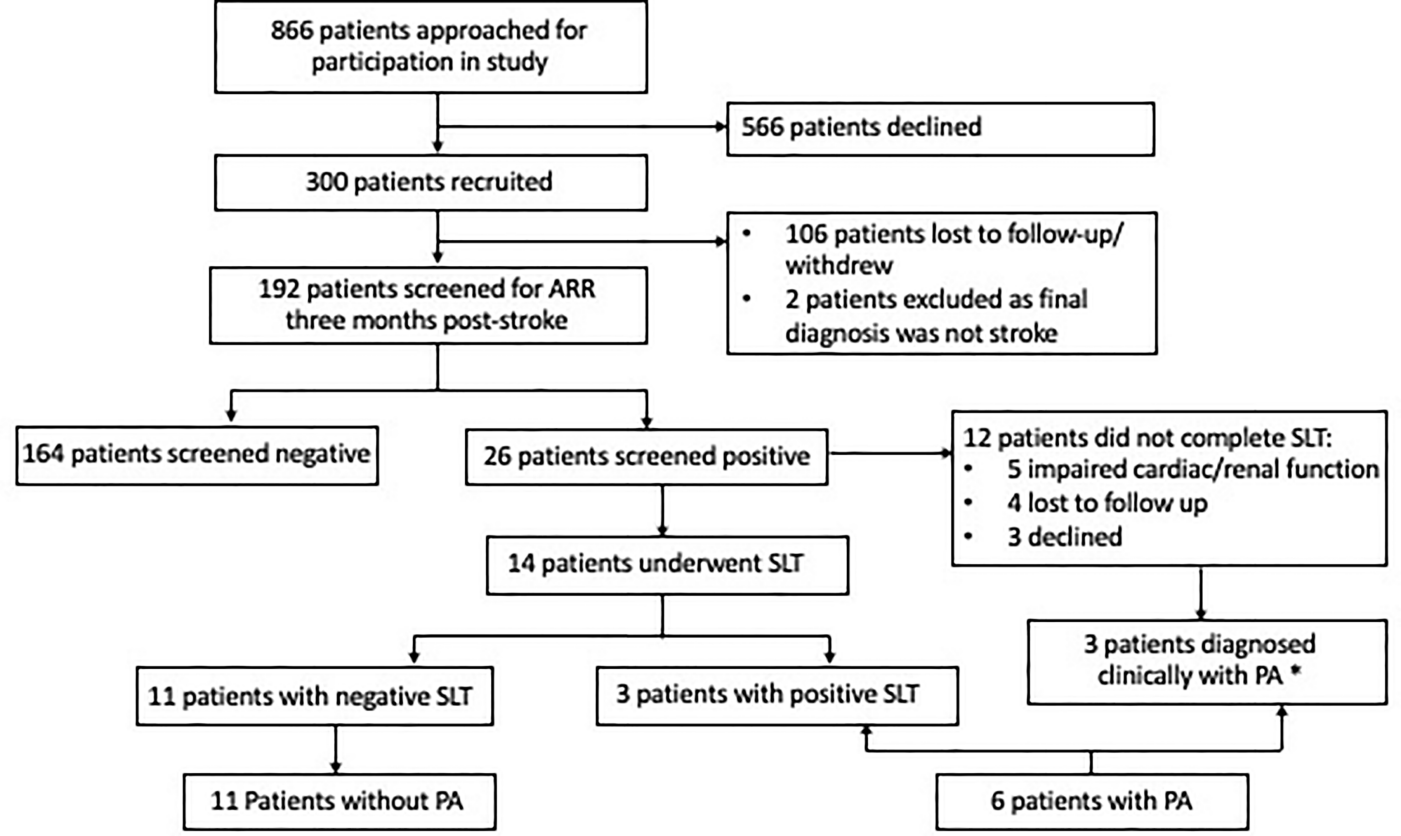
Consort diagram of 300 patients with acute stroke recruited. *One patient did not require confirmatory testing in view of the elevated aldosterone–renin ratio and spontaneous hypokalemia; two patients had elevated aldosterone and elevated aldosterone–renin ratio and were started on spironolactone. ARR, aldosterone–renin ratio; CVA, cerebrovascular accident; GFR, glomerular filtration rate; PA, primary aldosteronism; SLT, saline-loading test; TIA, transient ischaemic attack.

From August 2018 to October 2020, 886 eligible patients were admitted with the primary diagnosis of TIA or CVA to the acute stroke unit (**Figure 1**). We recruited 300 patients, of which two patients were subsequently excluded from analysis as their final clinical diagnosis was not stroke and 106 patients were lost to follow-up (41 patients were uncontactable, 22 patients were discharged to primary care, 35 patients withdrew consent, five patients went overseas, three patients died). At 3 months post-stroke, 192 patients underwent ARR screening, median age 58.0 years, with 137 (71.4%) females (**Table 1**). Amongst 192 patients, 150 (78.1%) had hypertension, 55 (28.6%) had diabetes, 60 (31.3%) had history of smoking, and 24 (12.5%) had a previous stroke. Stroke subtypes were ischaemic in 156 (81.3%), haemorrhagic in 20 (10.4%), and TIA in 16 (8.3%) patients. Patients who underwent ARR screening were slightly younger (58.0 vs. 61.0 years, *p* = 0.03) compared to those who did not, but otherwise there were no other significant differences between the two groups (**Supplementary Table 1**).

**Table 1:** Baseline characteristics of patients who completed the screening aldosterone–renin-ratio test.

Baseline characteristics	N = 192
***Demographics***	
Age, years	58.0 [21.0, 78.0]
Male	137 (71.4)
Body mass index, g/m^2^	25.5 [13.8, 45.7]; N = 183
Potassium, mmol/L	4.0 [2.9, 5.8]
Estimated glomerular filtration rate, mL/min/1.73 m²	94.6 [41.3, 130]
Systolic blood pressure, mmHg	157 [80, 247]; N = 187
Diastolic blood pressure, mmHg	90 [51, 142]; N = 187
Serum aldosterone, pmol/L	155 [38, 1,551]
Plasma renin activity, ng/ml/h	1.7 [0.6, 24.0]
Aldosterone–renin ratio, pmol/L per ng/ml/h	122 [6, 922]
***Comorbidities***	
Hypertension	150 (78.1)
Diabetes	55 (28.6)
Dyslipidaemia	192 (100)
History of smoking	60 (31.3)
History of drinking	34 (17.7)
Ischaemic heart disease	27 (14.1)
Atrial fibrillation	10 (5.2)
Chronic kidney disease	15 (7.8)
Stroke history	24 (12.5)
***Stroke subtypes***	**N (%)**
Ischaemic stroke	156 (81.3)
Small-artery occlusion	50 (26.0)
Large-artery atherosclerosis	25 (13.0)
Cardioembolic	19 (10.0)
Undetermined	62 (32.3)
Haemorrhagic stroke	20 (10.4)
Transient ischaemic attack	16 (8.3)
***Modified Rankin Scale Score***	1.2 (1.4); N = 183
***Use of antihypertensive medication***	
ACE-inhibitor or angiotensin II receptor blocker	69 (35.9)
Beta-blocker	46 (24.0)
Calcium channel blocker	73 (38.0)
Diuretics	2 (1.0)
Alpha blocker	1 (0.52)
Number of antihypertensive medication	1.0 (0.91)
***2D echocardiogram parameters***	
Left ventricular ejection fraction, %	60.0 [15.0, 66.0]; N = 169
Presence of left ventricular hypertrophy	9 (5.33%); N = 169
Relative wall thickness, h/r	0.4 [0.2, 0.7]; N = 165
Left atrium volume index, ml/m^2^	24.4 [13.2, 134]; N = 162
Left ventricular mass index, m^2^	69.8 [39.3, 173]; N = 164

Data presented as median [min, max], mean (SD), or number (%) as appropriate.

### Patients With Positive ARR

Twenty-six of 192 patients (14%) had a positive ARR screening test. The positive ARR group had higher diastolic blood pressure compared to the negative ARR group, 84.5 vs. 79.5 mmHg, *p* = 0.003, despite the similar number and type of antihypertensive medications (**Table 2**). Calcium-channel blockers and ACE inhibitors were the most commonly used. Patients with positive ARR were more likely to have AF, 19.2% vs. 3.0%, *p* = 0.005, and had greater left ventricular mass index (g/m^2^), 79.1 (69.8–99.8) vs. 67.8 (57.4–81.5), *p* = 0.02. There was no difference in stroke subtype, and MRS score post-stroke between the groups. We repeated this analysis after restricting only to patients with hypertension (**Supplementary Table 2**) since hypertension may independently increase the risk of AF and left ventricular mass. We similarly found that hypertensive patients with positive ARR were more likely to have AF, 5 (21.7%) vs. 4 (3.2%), *p* = 0.005, and had greater left ventricular mass index (g/m^2^), 84.7 (69.9–101.1) vs. 68.9 (57.9–85.4), *p* = 0.016.

**Table 2:** Characteristics of patients with positive ARR (N = 26) versus patients with negative ARR (N = 166).

Characteristics	Patients with ARR + N = 26 (13.5%)	Patients with ARR– N = 166 (86.5)	*p* value
***Demographics***			
Age, years	56.0 [47.5, 63.5]	58.0 [51.0, 64.1]	0.43
Male	16 (61.5)	121 (72.9)	0.25
Body mass index, g/m^2^	25.6 [25.0, 27.0]	25.5 [23.0, 28.3] N = 157	0.76
Systolic blood pressure, mmHg	145 [133, 148]	137 [125, 149] N = 162	0.11
Diastolic blood pressure, mmHg	84.5 [80.0, 92.0]	79.5 [74.0, 85.0] N = 162	0.003
Estimated glomerular filtration rate, mL/min/1.73 m²	94.3 [78.1, 100]	94.6 [82.5, 104]	0.56
Sodium, mmol/L	140 [139, 142]	140 [138, 141]	0.17
Potassium, mmol/L	3.9 [3.6, 4.1]	4.0 [3.7, 4.2]	0.25
Bicarbonate, mmol/L	23.0 [22.0, 25.8]	23.0 [21.0, 24.0]	0.26
Total cholesterol mmol/L	4.8 [4.1, 5.3]	4.7 [3.9, 5.4] N = 158	0.62
LDL mmol/L	3.2 [2.8, 3.8]	3.2 [2.6, 3.8] N = 158	0.73
HBA1c, %	5.9 [5.5, 6.7]	6.1 [5.5, 7.1] N = 161	0.37
Serum aldosterone, pmol/L	269 [213, 410]	139 [111, 222]	<0.001
Plasma renin activity, ng/ml/h	0.60 [0.60, 0.78]	1.95 [0.90, 3.38]	<0.001
Aldosterone-renin-ratio, pmol/L per ng/ml/h	355 [321, 452]	108 [47, 186]	<0.001
***Comorbidities***			
Hypertension	23 (88.5)	127 (76.5)	0.21
Diabetes mellitus	7 (26.9)	48 (29.3)	1.0
Dyslipidaemia	26 (100)	166 (100)	1.0
History of smoking	7 (26.9)	53 (31.7)	0.82
History of drinking	4 (15.4)	30 (18.3)	1.0
Ischaemic heart disease	4 (15.4)	23 (14.0)	0.77
Atrial fibrillation	5 (19.2)	5 (3.0)	0.005
Chronic kidney disease	3 (11.5)	12 (7.2)	0.44
Stroke history	1 (3.9)	23 (13.9)	0.21
***Stroke subtypes***			
Ischaemic stroke	23 (88.5)	133 (80.1)	0.78
Undetermined	9 (34.6)	53 (31.9)	
Others	14 (53.9)	82 (48.2)	
Haemorrhagic stroke	2 (7.7)	18 (10.8)	
Transient ischaemic attack	1 (3.9)	15 (9.0)	
***Modified Rankin Scale Score***	0.92 (1.3)	1.2 (1.4)	0.33
	N = 15	N = 159	
***Antihypertensive medication***			
ACE-inhibitor or Angiotensin II receptor blocker	8 (30.8)	61 (36.7)	0.66
Beta–blocker	8 (30.8)	38 (22.9)	0.46
Calcium channel blocker	12 (46.2)	61 (36.7)	0.39
Diuretics	1 (3.9)	1 (0.60)	0.25
Alpha blocker	1 (3.9)	0 (0)	0.14
Number of antihypertensive medication	1.2 (1.2)	1.0 (0.86)	0.75
***2D echocardiogram parameters***			
Left ventricular ejection fraction, %	60.0 [55.0, 60.0] N = 15	60.0 [55.0, 60.0] N = 144	0.92
Presence of left ventricular hypertrophy	3 (12.0) N = 15	6 (4.2) N = 144	0.13
Relative wall thickness, h/r	0.42 [0.35, 0.48] N = 15	0.40 [0.34, 0.45] N = 140	0.37
Left atrium volume index, ml/m^2^	25.7 [22.2, 30.0]	24.0 [19.7, 28.5]	0.25
Left ventricular mass index, m^2^	79.1 [69.8, 99.8] N = 25	67.8 [57.4, 81.5] N = 139	0.016

Data presented as median [min, max], mean (SD), or number (%) as appropriate.

ARR, aldosterone–renin ratio.

### Patients With PA

Fourteen patients proceeded with confirmatory SLT, with three patients having post-SLT PAC >138 pmol/l, consistent with diagnosis of PA (**Figure 1**). Of the 12 patients who did not proceed with the confirmatory SLT test, five patients had poor cardiac or renal function (which developed/worsened after recruitment) which were contraindications to SLT, four were lost to follow-up, and three declined. One patient had baseline aldosterone ≥554 pmol/l, undetectable PRA, and spontaneous hypokalaemia and did not require additional tests to confirm PA. One patient was not able to undergo SLT due to poor cardiac function, and in view of repeated elevated ARRs (PAC 15 and 13 ng/dl, with undetectable renin), the patient was treated with spironolactone. One final patient had an inappropriate PAC (7.9 ng/dl) and undetectable renin despite being on an ACE inhibitor. Due to newly diagnosed AF and impaired cardiac function, spironolactone was started with clinical improvement. In total, six patients had confirmed or likely PA. All six were treated with spironolactone and did not pursue subtype testing: one patient migrated, three patients declined surgery (two were >65 years), and two were subsequently diagnosed with cardiac or renal impairment which made them poor surgical candidates.

All six patients with PA had hypertension, three had AF, and two had hypokalaemia. Patients with PA had a higher diastolic BP at the 3-month follow-up (87.0 vs. 80.0 mmHg, *p* = 0.01) and were more likely to use calcium-channel blockers (83.5% vs. 36.6%, p = 0.03), and the median number of BP medications used was 1.7 vs. 1.0, *p* = 0.06 **(** **Table 3** **)**. Patients with PA were more likely to have AF (50% vs. 3.8%, p = 0.002) compared to those without PA. Patients with PA had a higher left ventricular mass index (g/m^2^), 90.7 vs. 69.2, *p* = 0.01, and were more likely to have left atrial dilatation (defined as left atrial volume index >34 ml/m^2^), 50.0% vs. 11.8%, *p* = 0.03.

**Table 3:** Characteristics of patients with primary aldosteronism (N = 6) versus patients without primary aldosteronism (N = 186).

Characteristics	Patients with PA N = 6	Patients without PA N = 186	*p* value
***Demographics***			
Age, years	57.3 [44.5, 65.9]	58.0 [51.0, 64.0]	0.91
Male *N (%)*	4 (66.7)	133 (71.5)	1.0
Body mass index, g/m^2^	24.8 [21.8, 27.4]	25.5 [23.0, 28.2] N = 177	0.58
Systolic blood pressure, mmHg	145 [138, 147]	137 [126, 149] N = 182	0.36
Diastolic blood pressure, mmHg	87.0 [84.3, 92.8]	80.0 [74.0, 86.0] N = 182	0.011
Estimated glomerular filtration rate, mL/min/1.73 m²	78.2 [66.1, 89.6]	94.9 [82.5, 103]	0.060
Serum aldosterone, pmol/L	416 [271, 521]	150 [111, 238]	0.0014
Plasma renin activity, ng/ml/h	0.65 [0.60, 1.15]	1.7 [0.7, 3.2]	0.048
Aldosterone–renin ratio, pmol/L per ng/ml/h	343 [319, 609]	116 [53, 188]	<0.001
***Comorbidities***			
Hypertension	6 (100)	144 (77.4)	0.34
Diabetes mellitus	2 (33.3)	53 (28.5)	1.0
Dyslipidaemia	6 (100)	186 (100)	1.0
History of smoking	2 (33.3)	58 (31.2)	1.0
History of drinking	0 (0)	34 (18.3)	0.59
Ischaemic heart disease	1 (16.7)	26 (14.0)	1.0
Atrial fibrillation	3 (50.0)	7 (3.8)	0.0019
Chronic kidney disease	1 (16.7)	14 (27.5)	0.39
Stroke history	0 (0)	24 (12.9)	1.0
***Stroke subtypes***			
Ischaemic stroke	6 (100)	150 (80.6)	1.0
Undetermined	2 (33.3)	60 (32.3)	
Others	4 (66.7)	90 (67.7)	
Haemorrhagic stroke	0 (0)	20 (10.8)	
Transient ischaemic attack	0 (0)	16 (8.6)	
***Modified Rankin Scale Score***	0.33 (0.82)	1.2 (1.4) N = 178	0.10
***Antihypertensive medication***			
ACE-inhibitor or angiotensin II receptor blocker	2 (33.3)	67 (36.0)	1.0
Beta-blocker	3 (50.0)	43 (23.1)	0.15
Calcium channel blocker	5 (83.3)	68 (36.6)	0.030
Diuretics	0 (0)	2 (1.1)	1.0
Alpha blocker	0 (0)	1 (0.5)	1.0
Number of anti-HTN meds, *mean*	1.7 (0.82)	1.0 (0.9)	0.06
***2D echocardiogram parameters***			
Left ventricular ejection fraction, %	52.5 [30.0, 60.0]	60.0 [55.0, 60.0] N = 163	0.11
Presence of left ventricular hypertrophy	0 (0)	9 (5.5) N = 163	1.0
Relative wall thickness, h/r	0.41[0.33, 0.42]	0.40 [0.35, 0.45] N = 159	0.61
Left atrium volume Index, ml/m^2^	31.2 [25.6, 44.0]	24.0 [19.8, 28.2]	0.032
Left ventricular mass index, m^2^	90.7 [83.9, 102]	69.2 [57.9, 83.7]	0.012

Data presented as median [min, max], mean (SD), or number (%) as appropriate.

PA, primary aldosteronism.

**Figure 2:**
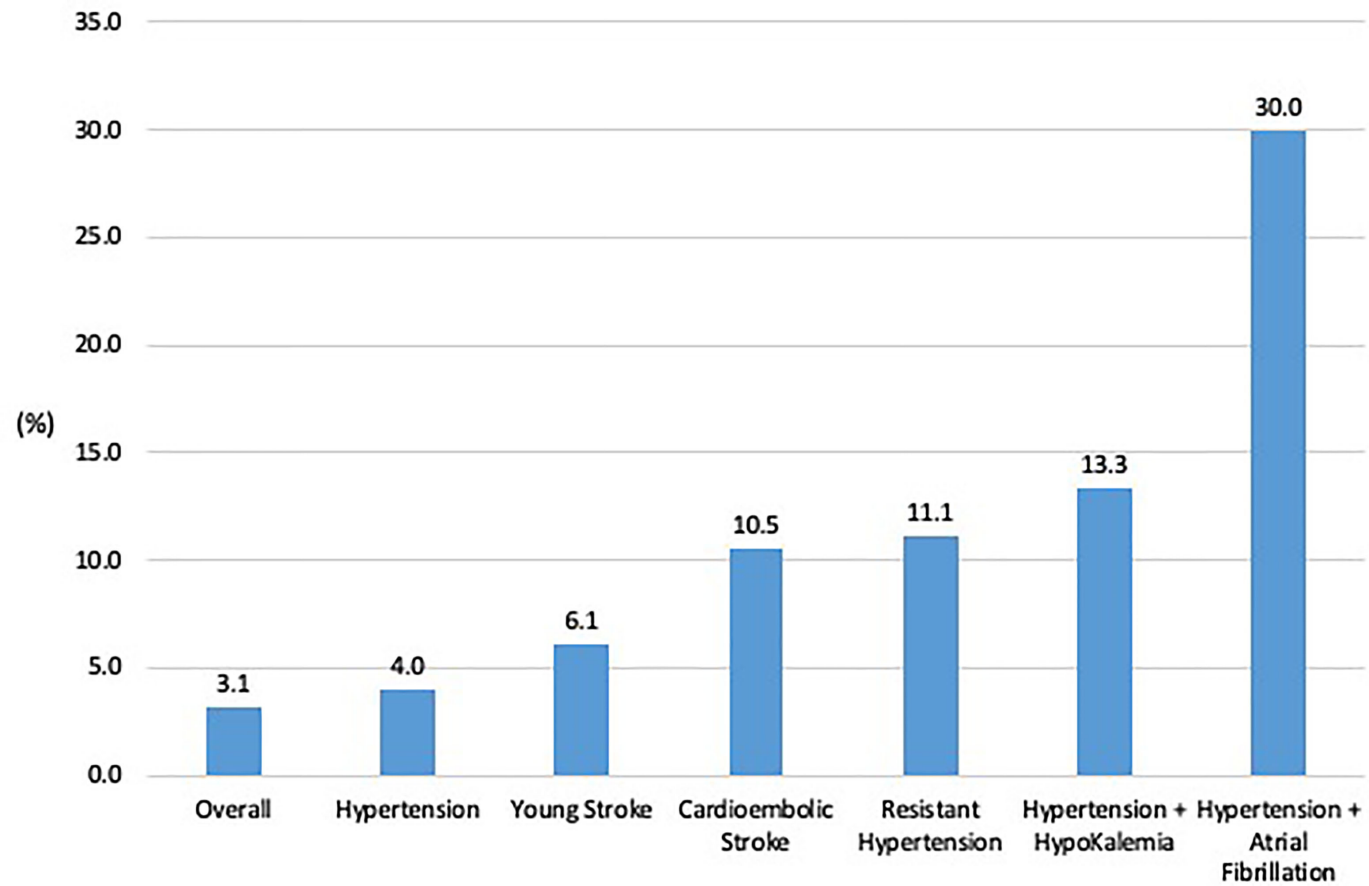
Prevalence rate of primary aldosteronism in the entire cohort and various subgroups.

The prevalence of PA in the cohort was 3.1%, 95% CI:1.2–6.7%, amongst all patients, and 4.0%, 95% CI: 0.9%–7.1%, amongst patients with hypertension (**Figure 2** **)**. Prevalence rates were higher in subgroups: age ≤50 years, 6.1% (3 of 49), 95% CI: 1.3%–16.9%, cardioembolic strokes, 10.5% (2 of 19), 95% CI: 1.3%–33.1%, resistant hypertension, 11.1% (1 of 9), 95% CI: 0.3%–48.3%, hypertension with hypokalaemia, 13.3% (2 of 15), 95% CI: 1.7%–40.5%, hypertension and AF, 30.0% (3 of 10), 95% CI: 6.7%–65.3%.

### Decision Tree Algorithm

**Figure 3:**
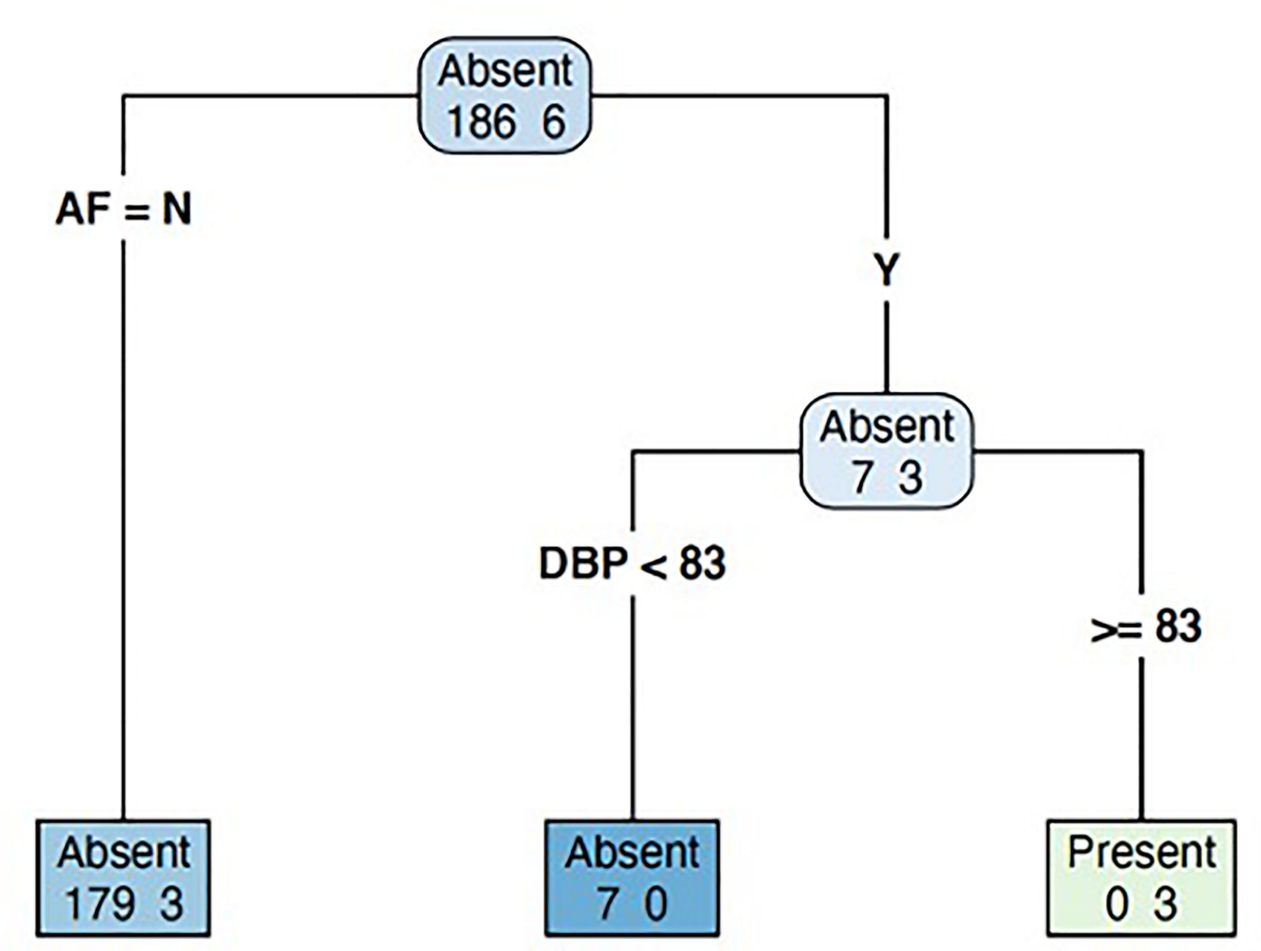
Decision tree model to predict the likelihood of primary aldosteronism in patients with recent stroke. AF, atrial fibrillation; DBP, diastolic blood pressure.

Our decision tree algorithm was derived from the 192 patients for whom complete data existed for all predictor and outcome variables, and it identified two main factors: diastolic BP and presence of AF. Patients with a diastolic BP ≥83 mmHg were more likely to have PA, and this increased to 100% amongst those with both diastolic BP ≥83 mmHg and presence of AF at 3 months post-stroke (**Figure 3**). In the 169 patients with 2D-echocardiography data, the decision tree algorithm yielded similar results.

## Discussion

In our prospective cohort study, PA was prevalent in at least 4% of hypertensive patients with stroke, which increased to 11% amongst those with cardioembolic stroke and 30% amongst those with atrial fibrillation. There has been a growing body of evidence that PA is associated with increased risk of AF (7, 13), and our study identifies another high-risk group of patients that should be screened for PA, namely those with cardioembolic stroke, or stroke with concomitant atrial fibrillation. We also found that patients with PA or elevated ARR had a greater left ventricular mass index, which highlights the deleterious effects of hyperaldosteronism, as well as importance of appropriate diagnosis and treatment of PA.

AF is the main predisposing factor for embolic strokes, which accounts for 15%–20% of all strokes (19). Excessive aldosterone is a major contributor to AF pathogenesis by inducing both structural cardiac remodelling through atrial dilatation and fibrosis, and electrical remodelling through arrhythmogenicity (20, 21). In our study, patients with positive ARR had higher rates of AF and greater left ventricular mass index compared to those with negative ARR, suggesting that the effects of hyperaldosteronism may not be restricted only to patients with PA, as has been previously reported (22). Patients with elevated ARR are particularly responsive to mineralocorticoid antagonists. Hence, patients with elevated ARR but normal response to saline suppression (false-positive ARR) may still benefit from therapy with spironolactone, similar to patients with PA (23). In addition, our patients with PA had a larger left atrial volume index, which predisposes to development of AF. Milliez and colleagues found a 12-fold higher risk of prevalent AF amongst patients with PA (6), compared to patients with essential hypertension, which has been supported by subsequent studies (24). Most recently, Seccia and colleagues found a high prevalence of PA in 42% of hypertensive patients with unexplained AF (8), which is not dissimilar from our prevalence of 30% amongst hypertensive patients with AF and stroke. Hence, our study confirms findings from previous studies that patients with PA are at a higher risk of stroke. Of particular note, one of our patients with PA was classified as embolic stroke of undetermined source (ESUS), because repeated cardiac monitoring (24-hHolter and extended 28-day Spyder) did not reveal any episodes of AF. ESUS may be attributed to various potential embolic sources, including occult atrial cardiopathy, and some patients may go on to develop AF (25). Hence, it is plausible that PA may be similarly common amongst patients with ESUS, and these patients should be offered screening for PA.

Hypertension is the main risk factor for haemorrhagic strokes, as well as ischaemic strokes from small- or large-vessel disease (9). Patients with PA have a 4–5-fold greater odds of stroke compared to patients with essential hypertension (6, 26), which is attributed to the deleterious effects of aldosterone on cerebral vasculature through oxidative stress and endothelial dysfunction (27). In our cohort, patients with either elevated ARR or PA had a greater left ventricular mass index, which underlines the effects of hyperaldosteronism on the myocardium (28). While all of our PA patients had hypertension, less than half had hypokalaemia, or were aged below 50 years, highlighting that hypokalaemia and age thresholds should not be prerequisites to screen for PA. One previous study on young stroke patients (below 45 years) found the prevalence rate of PA to be 12% (21). If we restricted screening to patients below 45 years of age, we would have only diagnosed one patient. Miyaji and colleagues found the prevalence of PA to be 4% amongst a cohort of patients with recent stroke, and 4.9% when restricted to those with hypertension, but did not report on the prevalence of AF (29). In their study, patients were screened for PA in the first 2 weeks post-stroke, which is not ideal as malignant hypertension or acute illness may alter PAC and PRA, and cosyntropin stimulation was used as a confirmatory test for PA, which is not currently recommended by guidelines (15).

Our findings suggest that all patients with previous stroke may benefit from screening for PA. Treatment of PA has been shown to reduce the risk of developing AF, cardiovascular, and cerebrovascular diseases (30). Although earlier studies found both surgical and medical therapy equally effective, more recent data suggest that surgical treatment leads to better outcomes (12, 13). None of our patients underwent subtype testing with adrenal vein sampling and surgery, similar to previous studies (29, 31), due to poor functional status or comorbidities in the patients. This emphasizes the importance of early diagnosis of PA, which unfortunately remains suboptimal, with less than 1% of all patients at risk of PA being screened (32). Early diagnosis and treatment of PA reduces target organ damage and increases the likelihood of cure of hypertension (33, 34). While patients diagnosed with PA post-stroke may not be good candidates for surgery, they are at high cardiovascular risk and can still benefit from aldosterone antagonists. Eplerenone treatment has been shown in animal models to reduce the aldosterone-induced damage to the cerebral cortex (35). In our retrospective cohort, 14 of our 154 (9%) patients with PA had a prior diagnosis of CVA (36). Hence, while there should be sustained efforts in the early diagnosis and treatment of PA, the event of a stroke should prompt clinicians to consider screening for secondary causes, particularly in patients with good functional recovery.

We recognize several limitations of our study. First, as our number of patients diagnosed with PA was small, we included 95% CI to reflect the strength of our prevalence estimates. Second, one-third of patients enrolled did not undergo a screening test. However, their baseline characteristics did not differ greatly from patients who proceeded with screening and are unlikely to affect our estimates. Third, not all patients with a positive screening test proceeded with a confirmatory SLT, due to cardiac or renal dysfunction. Hence, there are likely more patients with PA and a higher prevalence of PA. Fourth, a single PAC assessment may not fully reflect aldosterone status, and studies using 24-h urinary aldosterone have found a higher prevalence of PA (2). Finally, ARR can be affected by use of antihypertensive medications, such as ACE inhibitors and diuretics, leading to false-negative results (15). To reduce false-negative screening rates, we adopted a lower ARR threshold for case detection. Furthermore, other studies have similarly screened for PA with these medications on-board (1), and we had to avoid changing antihypertensive medications soon after a recent stroke as stringent BP control is of critical importance (37). Despite these known limitations when screening patients with stroke (38), we were able to diagnose a significant proportion of patients with PA. This underlies the fact that PA is common and often missed early on in its natural history. Following the call that more patients with stroke should be screened for PA (38), our study offers clinicians a feasible strategy to do so.

In conclusion, we found PA to be prevalent in patients with a recent stroke, similar to cohorts of hypertensive patients, and particularly higher in those with cardioembolic stroke, and concomitant AF. Restricting screening of PA to young patients or those with hypokalaemia would have missed a majority of patients with PA. Hence, we suggest screening all hypertensive patients with stroke for PA as a possible underlying cause, particularly if they have good functional recovery. The impetus for screening these patients is that appropriate diagnosis and treatment can ameliorate BP control, potentially identify a treatable and potentially curable cause of hypertension, and prevent a recurrent stroke, which may be catastrophic.

### Perspectives

Current guidelines recommend screening for PA in patients with severe or resistant hypertension, and hypertension with hypokalaemia. Our study supports the increasing call that presence of AF should be included as another indication for screening. In addition, patients with previous strokes are more likely to have PA. While these patients may not be ideal candidates for surgical treatment, they are at a high cardiovascular risk, and specific medical treatment for hyperaldosteronism is warranted. Early diagnosis and treatment for all patients with PA are the ultimate goal.

## Data Availability Statement

The raw data supporting the conclusions of this article can be made available by the authors upon request.

## Ethics Statement

The studies involving human participants were reviewed and approved by SingHealth Institutional Review Board. The patients/participants provided their written informed consent to participate in this study.

## Author Contributions

VN, SS, and TP had full access to the data in the study and take responsibility of the integrity of the data and accuracy of data analysis. TT, MM, JL, MZ, and TP recruited patients in the study and performed the data collection. TT, TA, LO, SF, MZ, and TP worked on the study concept and design. VN, TT, SF, and TP were involved in drafting of the manuscript. All authors contributed to the article and approved the submitted version.

## Funding

This research was supported by a grant from the Changi Health Fund (CHF2017.01-P and CHF2020.05-S to MZ).

## Conflict of Interest

The authors declare that the research was conducted in the absence of any commercial or financial relationships that could be construed as a potential conflict of interest.

## Publisher’s Note

All claims expressed in this article are solely those of the authors and do not necessarily represent those of their affiliated organizations, or those of the publisher, the editors and the reviewers. Any product that may be evaluated in this article, or claim that may be made by its manufacturer, is not guaranteed or endorsed by the publisher.
