# Supplementary material for: Primary Aldosteronism More Prevalent in Patients With Cardioembolic Stroke and Atrial Fibrillation
Source: Front Endocrinol (Lausanne). 2022 Apr 19;13:869980. doi: 10.3389/fendo.2022.869980 (PMC9063461; doi:10.3389/fendo.2022.869980)
Supplement: Supplementary file 1 [file DataSheet_1.pdf]

Supplementary Material

Supplementary Table 1. Ischemic Stroke Subtypes based on Trial of Org 10172 in Acute Stroke Treatment (TOAST)[1] classification.

Large-artery atherosclerosis (embolus/thrombosis)
Cardioembolism*
Small-vessel occlusion (lacune)†
Stroke of other determined etiology
Stroke of undetermined etiology<br>a. Two or more causes identified<br>b. Negative evaluation<br>c. Incomplete evaluation

*Permanent or paroxysmal atrial fibrillation, sustained atrial flutter, intracardiac thrombus, prosthetic cardiac valve, atrial myxoma or other cardiac tumours, mitral stenosis, recent (<4 weeks) myocardial infarction, left ventricular ejection fraction less than 30%, valvular vegetations, or infective endocarditis.

†Lacunar defined as a subcortical infarct smaller than or equal to 1.5 cm (≤2.0 cm on MRI diffusion images) in largest dimension, including on MRI diffusion-weighted images, and in the distribution of the small, penetrating cerebral arteries; visualisation by CT usually needs delayed imaging greater than 24–48 h after stroke onset.

Supplementary Table 2. Patients who underwent aldosterone-renin-ratio screening test (N=192) and those who did not undergo screening test (N=104)

Charateristics	**All patients** N=296	**All Patients with screening test** N=192	**All Patients with no screening test** N=104	***P* value**
Demographics				
Age, years	59.0 [51.0, 65.0]	58.0 [50.0, 64.0]	61.0 [53.0, 66.0]	0.030
Male	206 (69.6)	137 (71.4)	69 (66.3)	0.3
Body mass index, g/m^2	25.3 [22.7, 28.3] N=283	25.5 [23.0, 28.2] N=183	24.8 [22.3, 28.5] N=100	0.46
Systolic blood presssure, mmHg	137 [126, 149] N=188	137 [126, 149] N=188	NA	NA
Diastolic blood pressure, mmHg	81.0 [74.0, 86.3] N=188	81.0 [74.0, 86.3] N=188	NA	NA
Potassium, mmol/L	4.0 [3.7, 4.2]	4.0 [3.7, 4.2]	4.0 [3.7, 4.3]	0.39
Estimated glomerular filtration rate, mL/min/1.73 m^2	93.3 [79.6, 102]	94.6 [81.1, 103]	91.2 [77.5, 101]	0.18
Comorbidities				
Hypertension	229 (77.4)	150 (78.1)	79 (76.0)	0.67
Diabetes	96 (32.4)	55 (28.6)	41 (39.4)	0.069
Dyslipidemia	296 (100)	192 (100)	104 (100)	1.0
History of smoking	89 (30.1)	60 (31.3)	29 (27.9)	0.60
Ischemic Heart disease	47 (15.9)	27 (14.1)	20 (19.2)	0.25
Stroke history	43 (14.5)	24 (12.5)	19 (18.3)	0.23
Atrial fibrillation	16 (5.4)	10 (5.2)	6 (5.8)	0.80
Stroke subtypes				
Ischemic stroke	235 (79.4)	156 (81.3)	79 (76.0)	0.51
Large artery atherosclerosis	41 (13.9)	25 (13.0)	16 (15.4)	
Cardioembolism	29 (9.8)	19 (9.9)	10 (9.6)	

Small vessel occlusion	80 (27.0)	50 (26.0)	30 (28.8)	
Undetermined	85 (28.7)	62 (32.3)	23 (22.1)	
Haemorrhagic stroke	35 (11.8)	20 (10.4)	15 (14.4)	
Transient ischemic attack	26 (8.8)	16 (8.3)	10 (9.6)	
Modified Rankin Scale Score	1.00 [0, 2.00] N=256	1.00 [0, 2.00] N=184	0 [0, 2.00] N=72	0.55

Data presented as median [min, max], mean (SD), or number (%) as appropriate

Supplementary Table 3: Characteristics of hypertensive patients with positive ARR (N=23) versus hypertensive patients with negative ARR (N=127)

**Characteristics**	**Patients with ARR +** N=23 (15.3%)	**Patients with ARR-** N=127 (84.7)	***P* value**
Comorbidities/ Risk factors for stroke	**N (%)**		
Ischemic Heart diseases	4 (17.4)	18 (14.2)	0.7
Atrial fibrillation	5 (21.7)	4 (3.2)	0.005
Chronic Kidney Disease	3 (13)	12 (9.5)	0.7
Stroke subtypes	**N (%)**		
Ischemic stroke	20 (87)	103 (81.1)	1.0
Undertermined	7 (30.4)	40 (31.5)	
Others	13 (56.5)	63 (49.6)	
Hemorrhagic stroke	2 (8.7)	14 (11)	
Transient ischemic attack	1 (4.4)	10 (7.9)	
2D Echocardiogram Parameters	**Median [Q1,Q3]**		
Left Ventricular Ejection Fraction, %	60.0 [55.0, 60.0] N=22	60.0 [55.0, 60.0] N=108	0.9
Presence of Left Ventricular Hypertrophy *N(%)*	3 (13.6)	5 (4.6)	0.1
Relative Wall Thickness, h/r	0.42 [0.35, 0.48] N=22	0.40 [0.35, 0.46] N=105	0.6
Left Atrium Volume Index, ml/m^2	26.0 [22.2, 36.0] N=22	24.3[19.7, 29.2] N=105	0.2
Left Ventricular Mass index, m^2	84.7 [69.9, 101.1] N=22	68.9 [57.9, 85.4] N=105	0.016

Data presented as median [min, max], mean (SD), or number (%) as appropriate

References

1. Adams HP, Bendixen BH, Kappelle LJ, Biller J, Love BB, Gordon DL, et al. Classification of subtype of acute ischemic stroke. Definitions for use in a multicenter clinical trial. TOAST. Trial of Org 10172 in Acute Stroke Treatment. Stroke. 1993 Jan;24(1):35–41.
